# A Pan-Cancer Study of KMT2 Family as Therapeutic Targets in Cancer

**DOI:** 10.1155/2022/3982226

**Published:** 2022-01-11

**Authors:** Jiamin Zhu, Zhili Liu, Xiao Liang, Lu Wang, Dan Wu, Weidong Mao, Dong Shen

**Affiliations:** ^1^Department of Oncology, The Affiliated Jiangyin Hospital of Southeast University Medical College, Jiangyin 214400, China; ^2^Division of Colorectal Surgery, Department of General Surgery, The First Affiliated Hospital of Nanjing Medical University, Nanjing 210029, China

## Abstract

**Objective:**

Exome sequencing studies have shown that the histone-lysine N-methyltransferase 2 (KMT2) gene is one of the most commonly mutated genes in a range of human malignancies and is linked to some of the most common and deadly solid tumors. However, the connection between this gene family's function and tumor type, immunological subtype, and molecular subtype dependency is still unknown.

**Methods:**

We examine the expression patterns of the histone-lysine N-methyltransferase 2 (KMT2) gene, as well as their relationship to patient survival. We also used a pan-cancer analysis to link their function to immunological subtypes, the tumor microenvironment, and treatment sensitivity.

**Results:**

Using the TCGA pan-cancer data, researchers looked at and examined KMT2 expression patterns and their links to patient survival and the tumor microenvironment in 33 cancer types. The expression of the KMT2 family changes significantly across and within cancer types, indicating significant inter- and intracancer heterogeneity. Patients' overall survival was often linked to the expression of KMT2 family members. However, the direction of the link differed depending on the KMT2 isoform and cancer type studied. Notably, in all cancer types examined, nearly all KMT2 family members were substantially linked with overall survival in patients with renal clear cell carcinoma (KIRC). Furthermore, all KMT2 genes have a strong relationship with immune infiltrate subtypes, as well as varying degrees of stromal cell infiltration and tumor cell stemness. Finally, we discovered that higher expression of KMT2s, particularly KMT2F and KMT2G, was linked to greater chemotherapeutic sensitivity in several cell lines.

**Conclusions:**

The necessity to investigate each KMT2 member as a distinct entity inside each particular cancer type is highlighted by our comprehensive investigation of KMT2 gene expression and its relationship with immune infiltrates, tumor microenvironment, and cancer patient outcomes. Our research also confirmed the identification of KMT2 as a potential therapeutic target in cancer, but further laboratory testing is required.

## 1. Introduction

Epigenetics means that the DNA sequence does not change, but the gene expression changes heritably; that is, the genotype does not change, but the phenotype changes [1–3]. In other words, it is a way of inheritance outside of the DNA sequence. The conventional genetic information, which is given by DNA sequence, is found in the genome, whereas epigenetic information, which gives instructions on when, where, and how to apply genetic information, is found in the epigenome. Epigenetic regulation is the core of cellular characteristics in multicellular organisms [4]. Each cell type has its epigenome, which is divided into active, stable, and quiet areas by DNA methylation patterns and histone-specific modifications [5].

Histone-lysine N-methyltransferase 2(KMT2) family protein methylation lysine 4 in the tail of histone H3 is important for chromatin and DNA structural regulation [6]. Because of the function of KMT2A, the earliest member of the KMT2 family, the human KMT2 family was initially dubbed mixed-lineage leukemia (MLL) [7, 8]. However, recent exome sequencing studies have shown that the KMT2 gene is one of the most frequently mutated genes in a variety of human malignancies and that it is related to some of the most common and fatal solid tumors, including colon and lung cancer [9, 10]. Efforts to link KMT2's molecular processes to its involvement in carcinogenesis have resulted in the creation of first-generation inhibitors of KMT2 activity, which may lead to the development of new cancer treatments [11, 12]. Nevertheless, there is a lack of understanding of the KMT2 family in renal cell carcinoma. At present, only one literature reported that KMT2G may be a good prognostic marker of KIRC (kidney renal clear cell carcinoma) and can be used as a therapeutic target of KIRC [13]. At present, new treatment methods such as immunotherapy have been applied to a variety of diseases [14], including renal clear cell carcinoma. Therefore, understanding the role of KMT2 family in renal clear cell carcinoma will play a positive role in the new treatment.

In this research, we analyzed the patterns of expression in this gene family and its correlation with patient outcomes survival in 33 cancer tissues of cancers using pan-cancer TCGA results and related their expression to the tumor microenvironment and pharmacological activity.

## 2. Methods

### 2.1. TCGA Pan-Cancer Data

RNA seq (RNA SeqV2 RSEM), clinical outcomes, stemness score based on mRNA (RNAss) and DNA methylation (DNAss), and immunological subtypes may all be downloaded from the TCGA pan-cancer data via the Xena browser (https://xenabrowser.net/data%20page/). The 33 cancer types included in the TCGA pan-cancer data were ACC, BLCA, BRCA, CESC, CHOL, COAD, DLBC, ESCA, GBM, HNSC, KICH, KIRC, KIRP, LAML, LGG, LIHC, LUAD, LUSC, MESO, OV, PAAD, PCPG, PRAD, READ, SARC, SKCM, STAD, TGCT, THCA, THYM, UCEC, UCS, and UVM (Supplementary Table S1). The overall number of samples available for this research was 11057, with 45 cholangiocarcinoma samples and 1217 breast cancer samples available for each disease type. For such, 15 cancer types have no or fewer than 5 specific healthy samples, and only the remaining 18 types of cancer have been used to test how there are differences in gene expression relative to neighboring typical tumors using a linear mixed-effect model. The relationship between each KMT2 family member's gene expression (as a continuous variable) and overall patient survival was investigated in a survival study.

### 2.2. Analysis of the Microenvironment of the Tumor

The estimated stromal score and immune score were used to analyze the degree of immune and stromal infiltration in different malignancies [15]. Spearman correlation was used to see whether there was a link between KMT2 expression and those scores. Six immune subgroups were discovered to evaluate immunological infiltrates in the tumor environment [16]. Using immune subtypes obtained from TCGA pan-cancer data, the Kruskal test was used to evaluate the association between KMT2 expression and immune infiltration types in the tumor microenvironment. Researchers used attributes obtained from transcriptomic and epigenetic data from TCGA tumor samples to determine if tumor cells had stem cell-like properties [17]. The association between cancer stemness and KMT2 expression was investigated using the Spearman correlation test.

### 2.3. Analysis of NCI-60

We used the Cell Miner software (https:/discover.nih.gov/cellminer/) to access the NCI-60 database including information from nine tumor types in 60 different cancer cell lines. Cell-responsiveness results (GI50) from KMT2 mRNA expression and z values were obtained for 59 cell lines. The Pearson correlation coefficient was utilized to assess the link between gene expression and drug sensitivity. Drug responses from 262 FDA-approved or clinically studied medications were included in the correlation study [18].

### 2.4. Statistical Analyses

A gene expression analysis between regular and tumors was carried out in 18 forms of cancer, with more than 5 linked neighboring typical samples using linear longitudinal mixed-effect models. Gene expression in several kinds of cancer was shown using box diagrams. The researchers utilized Kaplan–Meier and Cox proportional hazard regression models to investigate the link between overall survival and gene expression. Spearman or Pearson correlations were utilized to examine the relationship between gene expression and stemness levels, stromal performance, immune score, estimation value, and drug susceptibility. The connection between gene expression and clinical characteristics of people, immunological components, and the stage of KIRC cancer is evaluated using linear regressions. All tests were performed using R software (v3.6.1) with packages ggpubr, ggplot2, pheatmap, corrplot, survival, survminer, limma, reshape2, estimate, or impute where appropriate [19].

## 3. Results

### 3.1. Expression of the KMT2 Gene in Various Cancers

**Figure 1:**
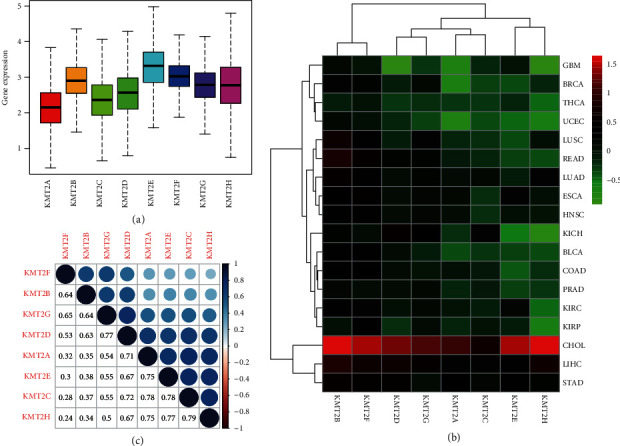
Expression levels of KMT2 genes in cancerous and adjacent normal tissues. (a) Boxplot to show the distribution of KMT2 gene expression across all 33 cancer types. (b) Heatmap to show the difference of KMT2 gene expression comparing primary tumor to adjacent normal tissues based on log2 (fold change) for 18 cancer types that have more than 5 adjacent normal samples. (c) Correlation plot based on Spearman correlation test results to show the correlation of gene expression among the 8 KMT2 family members across all 33 cancer types.

To further understand their intrinsic expression pattern, we looked at the levels of expression of KMT2 family members in all cancer types available in TCGA pan-cancer data. A significant intra- and intertumor heterogeneity concerning the expression rates of the related genes was found across all 8 KMT2 members (Figure 1(a) and Supplementary Figure 1). When it comes to the gene expression of a particular KMT2 family member, there was significant variability of each gene expression across various tumor types, with some tumor types expressing a very high level of a given gene and others exhibiting little heterogeneity. For example, all KMT2 family members showed a tendency of low expression in LICH and high expression in ESCA. These data all suggest that we need to study this gene family as a whole.

Dysregulated expression in cancer tumors is a distinguishing property of genes that function during tumorigenesis. In this study, we looked at the expression levels of all 8 genes in primary patient tumors from 18 different cancer types, as well as at least 5 matched normal samples (Figure 1(b)). Different cancer types revealed substantial differential expression of all KMT2, although the direction of the changed expression differed for each gene and cancer type (Figure 1(b)). With a few exceptions, KMT2B and KMT2F were mostly elevated in the malignancies studied. Furthermore, while the expression levels of various KMT2 family members were positively correlated with each other averaged across cancer types using Spearman correlation tests, we discovered that the pairs KMT2C and KMT2A (*r* = 0.78, *P* < 0.0001) and KMT2C and KMT2E (*r* = 0.78, *P* < 0.0001) had the highest correlation among all the pairwise correlations of the 8 genes, implying that they may share some common features or functions (Figure 1(c)).

### 3.2. Expression of the KMT2 Gene Is Linked to Patient Survival

**Figure 2:**
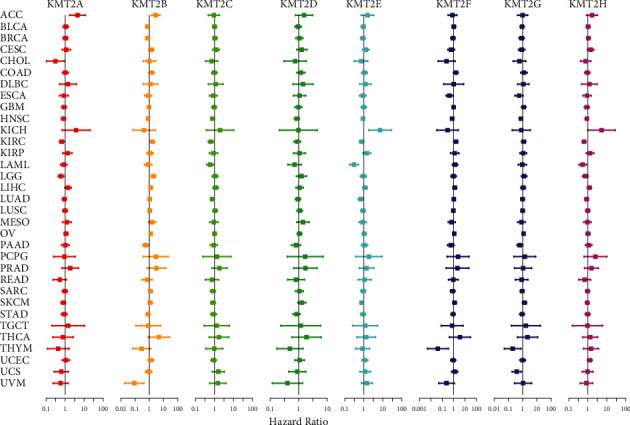
Association of KMT2 gene expression with patient overall survival for different cancer types. The forest plots with the hazard ratios and 95% confidence intervals for overall survival for different cancer types to show survival advantage and disadvantage with increased gene expression of KMT2 family. Univariate Cox proportional hazard regression models were used for the association tests.

The 33 tumors' survival data were used to examine the relationship between KMT2 gene expression and patient overall survival to correlate and predict whether KMT2 family members promote or hinder carcinogenesis in particular cancer types. We used the R-language survival package for the Cox proportional hazard regression model analysis and forest mapping, and we claimed that *P* < 0.05 was significantly related. We discovered that variations in KMT2s expression were linked to patient survival in general. The direction of this relationship, as shown in Figure 2, differs by member and kind of cancer diagnosed. Increased expression of KMT2C and KMT2G, in particular, was linked to a higher survival advantage, with KMT2C predicting a favorable prognosis in patients with KIRC, LAML, LUAD, and HNSC, and KMT2G predicting a good prognosis in patients with UCS, THYM, and HNSC. Depending on the kind of cancer, the remaining KMT2s are linked to survival benefits or drawbacks. KMT2A, in particular, indicated a bad prognosis for ACC but a better prognosis for LGG, KIRC, and CHOL. For LGG, ACC, and KIRC, KMT2B indicated a dismal prognosis, while for UVM, it projected a survival advantage. KMT2E predicted poor prognosis of KICH but predicted survival advantages of LAML, SKCM, LUAD, and KIRC. KMT2F indicated that KIRC would have a bad prognosis, but that THYM and ESCA would have a better prognosis. KMT2H indicated that KICH would have a bad prognosis, but that KIRC and LAML would have a better chance of surviving. Notably, nearly every member of the KMT2 family was shown to be related to overall survival in patients with renal clear cell carcinoma (KIRC) in all cancer types studied (*P* < 0.05) (Supplementary Figure 2). However, the direction of the connection is determined by the gene. Given that genes from the same family may be linked, a multivariate Cox proportional hazard regression model was employed to see whether significant connections remained after all members of the family were included in the model. KMT2A, KMT2B, KMT2C, KMT2E, and KMT2F remained substantially linked to patient survival, according to the findings (Figure 2).

### 3.3. KMT2 Genes Are Linked to the Immune System and the Tumor Microenvironment

**Figure 3:**
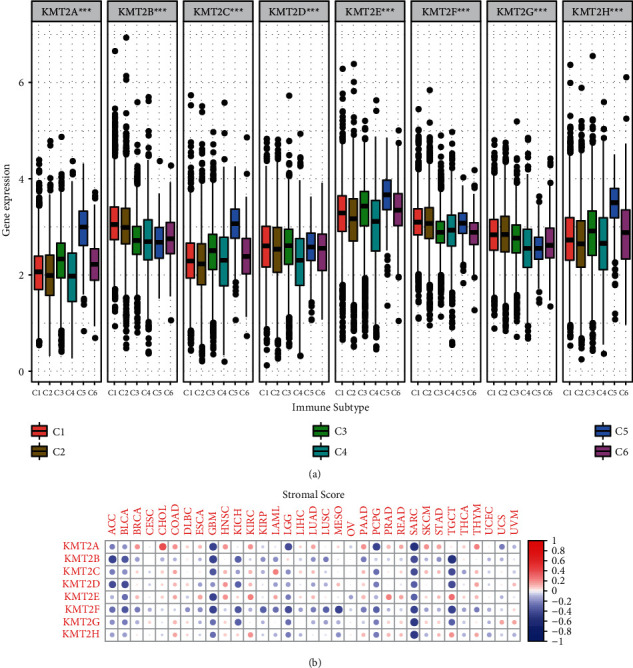
Association of KMT2 gene expression with tumor microenvironment factors. (a) Association of KMT2 gene expression with immune infiltrate subtypes across all the cancer types (*P* < .0001) tested with ANOVA. (b) Correlation matrix plots to show the association between KMT2 gene expression and stromal scores of 33 different cancer types based on ESTIMATE algorithm. Spearman correlation was used for testing. The size of the dots stands for the absolute value of the correlation coefficients. The bigger the size is, the higher the correlation is (higher absolute correlation coefficient).

In human cancer, a problem or mutation in the KMT2 family may alter the epigenetic properties of cells, resulting in disease onset and progression. Epigenetic effects have recently been shown to control the formation of blood cells in the immune system, which may be intimately linked to the immune system [20–22]. To better comprehend the relationship between KMT2 family members and immune components, researchers looked into the link between KMT2 and tumor immune invasion. In human malignancies, six different kinds of immune infiltration have been discovered, each of which corresponds to tumor promotion or tumor repression [23]. They are C1 (wound healing), C2 (INF-r dominant), C3 (inflammation), C4 (lymphocyte failure), C5 (Immune quiet), and C6 (TGF-*β* predominance). According to studies, patients with C3 and C5 immune subtypes had substantially better overall survival rates than those with other immune subtypes, whereas patients with C4 and C6 had the lowest overall survival rates of all cancer types [23]. High levels of KMT2A, KMT2C, KMT2E, and KMT2H were shown to be strongly connected with types 3 and 5 infiltrations (C3 and C5), indicating that greater gene expression is linked to a healthy immune system and that these genes may play an important function in tumor suppression. In contrast, the expression of KMT2B and KMT2G in C6 components was higher than that in C5, suggesting that these genes may act as tumor promoters (Figure 3(a)).

Using the ESTIMATE method, we looked at the relationship between KMT2 expression levels and the presence of infiltrating stromal cells in tumors that were indicated by stromal scores (Figure 3(b)) [15, 24]. Significant variations in the degree of connection between members of the KMT2 family and interstitial scores of various cancer kinds were also discovered. KMT2B had the strongest (*r* = −0.51, *P*.0001) association with cancer type stromal score, followed by KMT2A (*r* = −0.49, *P* < 0.0001) and KMT2G (*r* = −0.45, *P* < 0.0001). GBM, SARC, and TGCT were the cancers with the greatest association between various KMT2 family members and stromal scores. More specifically, we found that KMT2E was substantially adversely associated with the stromal score in GBM; KMT2A, KMT2G, and KMT2H were strongly negatively correlated with the stromal score in SARC; and KMT2B, KMT2C, KMT2D, and KMT2F were significantly negatively correlated with the stromal score in TGCT (*P* < .0001). We also looked at the relationship between KMT2s and immunization and assessment scores in the tumor, which evaluated the degree of immune cell infiltration and tumor purity, and found that the stromal score test produced comparable findings.

### 3.4. KMT2 Genes Are Linked to Tumor Stemness and Chemotherapy Sensitivity in Cancer Cells

**Figure 4:**
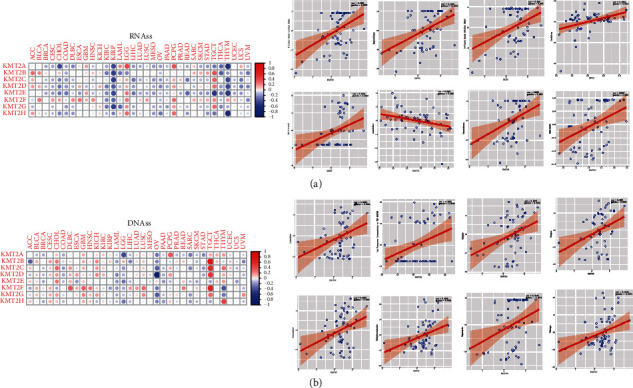
Association of KMT2 gene expression with tumor stemness and drug sensitivity. (a, b) Correlation matrix between KMT2 gene expression and cancer stemness scores RNAss (a) and DNAss (b), respectively, based on Spearman correlation tests. (c) Scatter plots to show the association between KMT2 gene expression and drug sensitivity (Z-score from Cell Miner interface) tested with Pearson correlation using NCI-60 cell line data.

Tumor cells progressively lose their differentiated phenotypes and gain progenitor and stem-like properties as cancer develops. The RNA stemness score (RNAss), which is based on DNA methylation pattern (DNAss) and mRNA expression, may be used to determine tumor stemness [17]. The researchers looked at the link between the KMT2 genes and tumor stemness as measured by RNAss and DNAss (Figures 4(a) and 4(b)). The members of the KMT2 family display varying rates of interaction with RNAss and DNAss in various forms of cancer. KMT2A, KMT2D, and KMT2H genes were shown to be negatively associated with RNAss and DNAss (*P* < 0.0001), with KMT2H having the highest connection (*r* = −0.57) with RNAss. Other KMT2 family members (KMT2B, KMT2C, KMT2E, KMT2F, and KMT2G) had minimal (*P*-value significant) or nonsignificant correlation coefficients with RNAss, but were highly associated with DNAss (*P* < 0.0001). All members of the KMT2 family were substantially associated with RNAss in LGG, KIPR, and TCGT, except for KIRP, which was negatively connected; the other two were positively correlated and negatively correlated. It should be noted that, in LGG, except KMT2B, all genes have a significant positive correlation with RNAss, while on the contrary, all genes except KMT2B have a significant negative correlation with DNAss. Not all genes in KIRP have a favorable correlation with DNAss. These contradictory results suggest that RNAss and DNAss may recognize different cancer cell groups with different characteristics or stemness degrees in different cancers.

We next looked at the expression of the KMT2s gene in 60 human tumor cell lines (NCI-60), which are susceptible to over 200 chemotherapeutic agents, and rigorously identified the connection between its expression levels in 60 human tumor cell lines (NCI-60). The greatest expression of KMT2E was found in all cancer cell lines, whereas the lowest expression was found in KMT2B. The Z-score technique was used to determine drug sensitivity. The more responsive the cells were to pharmacological treatment, the higher the score was. We discovered that higher expression of KMT2s, particularly KMT2F and KMT2G, was related to enhanced chemotherapeutic sensitivity of various cell lines (*r* > 0.4, *P* < 0.001) (Figure 4(c)). For example, KMT2F is associated with the cellular sensitivity of 5-fluorodeoxyuridine (treatment of breast cancer, gastric cancer, and colorectal cancer). In addition, KMT2G is associated with the cell sensitivity of temsirolimus (treatment of renal cell carcinoma). We also discovered that certain genes are linked to drug resistance to a variety of medicines. Furthermore, various genes may have inverse relationships with the same medication. KMT2E, for example, has been linked to greater cell resistance to fluorouracil (a medication used to treat malignancies of the stomach, colon, esophagus, rectal, and liver), while KMT2G has been linked to enhanced cell sensitivity to the same drug.

### 3.5. KMT2 Gene Family in Kidney Renal Clear Cell Carcinoma

**Figure 5:**
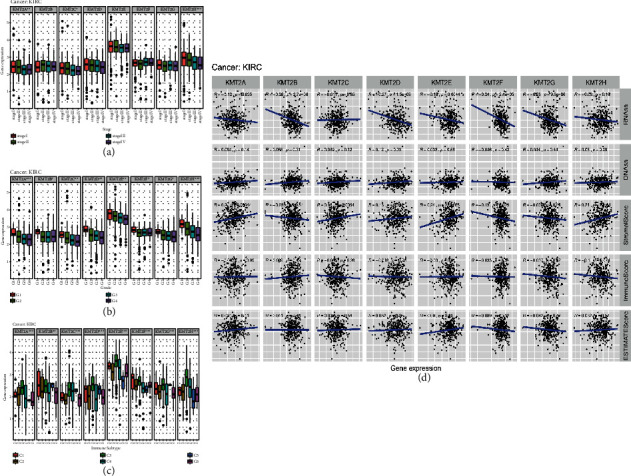
KMT2 gene expression in kidney renal clear cell carcinoma. (a) Association of KMT2 gene expression with kidney renal clear cell carcinoma molecular subtypes (*P* < .0001) was tested with ANOVA. (b) Association of KMT2 gene expression with immune infiltrate subtypes in kidney renal clear cell carcinoma tested with ANOVA (*P* < .0001). (c) Correlation matrixes between KMT2 gene expression and RNAss, DNAss, stromal score, immune score, and estimate score. Spearman correlation tests were used for testing.

We used TCGA kidney renal clear cell carcinoma data to thoroughly investigate the KMT2 gene in a cohort of renal cancer patients. Except for KMT2A and KMT2D, the expression of KMT2s in KIRC was significantly different (*P* < 0.001), and the expression of KMT2C and KMT2H was significantly decreased (Supplementary Figure 3). We also looked at how KMT2s were expressed at various phases of KIRC. The expression of KMT2A, KMT2C, and KMT2H decreased significantly with the progression of the disease in KIRC. Other KMT2s members also have a downward trend, but there is no significant difference (Figure 5(a)).

Another possible mechanism of controlling the expression of KMT2 in a tumor is that KMT2 is expressed differently in different types of cells in the tumor microenvironment. In renal clear cell carcinoma, the connection between KMT2 gene expression and immune subtypes was identical to that seen in all 33 TCGA cancers, and 8 genes were substantially related with the type of immune invasion (*P* < 0.001) (Figure 5(b)). We looked at the relationship between KMT2 expression and stromal score since stromal cells make up a significant component of the tumor microenvironment. We found that KMT2A, 2C, 2D, 2E, and 2H were positively correlated with the stromal score of renal clear cell carcinoma (*P* < 0.05), and KMT2E and KMT2H had the strongest correlation (*R* > 0.2). There was no significant correlation between KMT2B and KMT2G, but a negative correlation between KMT2F and interstitial score. It is suggested that KMT2A, 2C, 2D, 2E, 2F, and 2H may be expressed in the interstitium of renal clear cell carcinoma. Except for KMT2C and KMT2H, KMT2 family members were negatively correlated with RNA stemness score (*r* = −0.011 to −0.26, *P* < 0.05) and had little association with DNAss (*P* > 0.05) (Figure 5(d)).

## 4. Discussion

Histone 3 lysine 4 methylation (H3K4me) regulates a variety of cellular activities including replication, DNA damage response, cell cycle progression, and gene transcriptional control [25, 26]. Previous studies have shown that the downregulation of H3K4me is associated with a variety of human diseases, including tumors. Histone methyltransferases (HMTS) are also known as lysine methyltransferases (KMTs). Changes in different histone methylases have been linked to therapeutic and prognostic potential in human cancers, according to recent research [27, 28].

In this study, the histone-lysine N-methyltransferase 2 (KMT2) family was systematically analyzed by pan-cancer for the first time. We discovered a lot of variation in KMT2 gene expression levels across and among tumor types. KMT2G has the most intertumor heterogeneity out of all of them. Furthermore, in 18 types of cancer, KMT2B and KMT2F were mostly upregulated, whereas the other KMT2s were primarily downregulated. We next looked at the link between KMT2s expression and patient overall survival rates in 33 cancer types and discovered that the direction of the link was likewise cancer type dependent. However, in general, KMT2C and KMT2G were mainly related to better survival rate and KMT2D was not related to survival rate, while the other KMT2s were antagonistic to survival rate (both advantage and disadvantage). Our research discovered that all members of the KMT2 family were linked with subtypes of immune invasion in the tumor microenvironment, with KMT2B and KMT2F being associated with more aggressive subtypes of immune invasion, notably C1, C2, and C6, and poor prognosis. Based on the ESTIMATE methodology, KMT2s were also linked to stromal cell infiltrates and immune cell infiltrates to varying degrees. Epigenetic control seems to impact all cancer indicators, including all elements of tumor cell-immune system interactions. Therefore, epigenetic regulation can induce an antitumor immune response. Epigenetic treatment is often considered to be a modern form of immunotherapy.

The expression levels of KMT2A and KMT2C, as well as KMT2C and KMT2H, were the most associated across all 33 cancer types among the eight KMT2 gene members, indicating that they may share certain activities. We discovered that they were not only reduced in the 18 cancer types studied, but that they also had similar patterns associated with the immunoinfiltrative subtype, where they were highly expressed in immune subtypes with low proliferation rates (i.e., C3 and C5), implying the role of tumor inhibitors. KMT2C and KMT2H expressions were not substantially associated with RNAss, despite other members of the KMT2s family being strongly negatively correlated with tumor stem-like features assessed by mRNA (RNAss). Rather, they are related to tumor treatment resistance and have a positive relationship with the tumor immune score matrix score, suggesting that they may play an important role in tumor immunity. KMT2A has been researched the most, and there is increasing evidence that it plays a unique function in cancer formation. KMT2A was shown to be a dominant cancer gene affected by recurrent translocations in leukemias [29]. Recent studies have shown that KMT2A may play a recessive role in some solid tumors, such as gastric cancer. In gastric cancer, KMT2A is recruited into the ANO1 promoter to induce H3K4 methylation of the ANO1 promoter to activate ANO1. In gastric cancer patients, higher levels of ANO1 expression were linked to tumor spread [30]. However, we discovered that KMT2A expression was up- or downregulated in several cancer types and that KMT2A expression was linked with a favorable prognosis in certain cancer types (LGG, KIRC, and CHOL), while KMT2A expression was reduced in tumors. Therefore, the role of KMT2A as a tumor promoter needs to be reassessed. KMT2C, also known as MLL3 in humans, is a tumor suppressor associated with leukemia and other solid tumors that has histone methylation activity for transcriptional synergistic activation [31, 32]. However, in esophageal squamous cell carcinoma, KMT2C inhibits tumor growth [33]. In our study, KMT2D was upregulated or downregulated in 33 tumors. It is reported that KMT2D plays a role in the development of acute myeloid leukemia. Moreover, KMT2D plays a tumor inhibitory role in melanoma, pancreatic cancer cells, and lung cancer. These findings suggest that the antitumor or protumor effect of KMT2D may be related to cell type [34]. KMT2E has been suggested to lack intrinsic methyltransferase activity that is a common characteristic of the KMT2 family. However, a slew of evidence suggests that KMT2E may be involved in cancer tumor inhibition in certain kinds of subtypes [35]. In our study, KTM2E was also downregulated in the vast majority of cancers. KMT2H, also known as ASH1L, also encodes the histone-lysine methyltransferase. In breast cancer, ASH1L is often amplified at high levels, and high mRNA levels are linked to a lower life expectancy. Furthermore, HCC had a high level of ASH1L expression [36]. However, in our study, ASIL expression was decreased in most cancers, and in KIRC, decreased mRNA expression was associated with prolonged survival.

Compared with KMT2A, KMT2C, KMT2D, KMT2E, and KMT2H, other members of the KMT2s family showed significant upregulation in most tumors. They had a negative relationship with cancer stem cell-like RNAss, but a positive relationship with DNAss. KMT2B is known to be associated with infantile leukemia and tumor cell proliferation [37]. KMT2F and KMT2G are also known as SETD1A and SETD1B. SETD1B has recently been identified as a putative tumor suppressor gene associated with colorectal cancer and endometrial cancer. This means it has a strong role in cancer. In contrast, SETD1A has not been widely studied in cancer. In addition, we found that, unlike other members of the family, KMT2B and KMT2G expressed significantly C3 and C5 in immune infiltrated C6, and the immune subtype C6 was associated with reduced survival. Furthermore, KMT2F and matrix score have a significant relationship, indicating that they are released by stromal cells or engage in matrix-related activities.

CSCs (cancer stem-like cells) may be derived from a variety of sources, such as long-lived stem cells or progenitor cells, or by dedifferentiating cancer cells from nonstem cells. Deregulation of associated signaling pathways transforms these cells into CSCs. Cancer development is aided by CSCs' capacity to self-renew and infiltrate, which is the primary source of treatment-induced drug resistance.

We used RNAss and DNAss to investigate the expression of KMT2s with stem cell-like features. As previously stated, the KMT2A, KMT2D, and KMT2H genes were shown to be adversely associated with RNAss and DNAss. The correlation coefficients of other KMT2 family members (KMT2B, KMT2C, KMT2E, KMT2F, and KMT2G) were positively correlated with DNAss. We discovered that higher expression of KMT2s, particularly KMT2F and KMT2G, was linked to greater chemotherapeutic sensitivity of several cell lines. We also note that some genes are associated with drug resistance to several drugs. These results suggest that KMT2s may be involved in cancer cell drug sensitivity or resistance and that they may be used as therapeutic targets to overcome drug-induced resistance or enhance sensitivity.

## 5. Conclusion

The expression profile of KMT2 family genes was thoroughly and systematically characterized in this research, as well as its function in tumor type, immunological subtype, and molecular subtype dependency. To summarize, our findings indicate that KMT2A, KMT2C, KMT2D, and KMT2H are more likely to inhibit tumor occurrence, whereas other KMT2s are more likely to promote tumor occurrence and are generally associated with poor prognosis. The potential tumor promoter or antitumor impact of KMT2 is not consistent across family members in a particular cancer type, and the function of the KMT2 subtype is varied in various cancer types, even in distinct cancer subtypes. Finally, our findings will aid in the discovery of their function in carcinogenesis, particularly in immune response, tumor microenvironment, and drug resistance, all of which are critical for the creation of tailored cancer therapy medicines.

## Data Availability

The datasets involved in our study are publicly available in TCGA database (https://portal.gdc.cancer.gov/).

## Supplementary Materials

Supplementary Materials Supplementary Table 1: the 33 cancer types included in the TCGA pan-cancer data. Supplementary Figure 1: the levels of expression of KMT2 family members in all cancer types available in TCGA pan-cancer data. Supplementary Figure 2: overall survival in patients with renal clear cell carcinoma (KIRC) of KMT2 family members. Supplementary Figure 3: the expression of KMT2s in KIRC. s

## References

[1] Strahl B. D., Allis C. D. (2000). The language of covalent histone modifications. *Nature*.

[2] Jenuwein T., Allis C. D. (2001). Translating the histone code. *Science*.

[3] Lu L., Hu Y., Wang C., Jiang F., Wu C. (2021). Methylation and expression of the exercise-related TLR1 gene is associated with low grade glioma prognosis and outcome. *Frontiers in Molecular Biosciences*.

[4] Fontecha-Barriuso M., Martin-Sanchez D., Ruiz-Andres O. (2018). Targeting epigenetic DNA and histone modifications to treat kidney disease. *Nephrology Dialysis Transplantation: Official Publication of the European Dialysis and Transplant Association—European Renal Association*.

[5] Ruiz-Andres O., Sanchez-Niño M. D., Moreno J. A. (2016). Downregulation of kidney protective factors by inflammation: role of transcription factors and epigenetic mechanisms. *American Journal of Physiology-Renal Physiology*.

[6] Rao R. C., Dou Y. (2015). Hijacked in cancer: the KMT2 (MLL) family of methyltransferases. *Nature Reviews Cancer*.

[7] Shilatifard A. (2006). Chromatin modifications by methylation and ubiquitination: implications in the regulation of gene expression. *Annual Review of Biochemistry*.

[8] Schuettengruber B., Martinez A. M., Iovino N., Cavalli G. (2011). Trithorax group proteins: switching genes on and keeping them active. *Nature Reviews Molecular Cell Biology*.

[9] Ding L., Getz G., Wheeler D. A. (2008). Somatic mutations affect key pathways in lung adenocarcinoma. *Nature*.

[10] Cancer Genome Atlas N. (2012). Comprehensive molecular characterization of human colon and rectal cancer. *Nature*.

[11] Bernt K. M., Zhu N., Sinha A. U. (2011). MLL-rearranged leukemia is dependent on aberrant H3K79 methylation by DOT1L. *Cancer Cell*.

[12] Daigle S. R., Olhava E. J., Therkelsen C. A. (2011). Selective killing of mixed lineage leukemia cells by a potent small-molecule DOT1L inhibitor. *Cancer Cell*.

[13] Kumar A., Kumari N., Rai A., Singh S. K., Kakkar N., Prasad R. (2018). Expression and clinical significance of COMPASS family of histone methyltransferases in clear cell renal cell carcinoma. *Gene*.

[14] Jiang F., Wang X. Y., Wang M. Y. (2021). An immune checkpoint-related gene signature for predicting survival of pediatric acute myeloid leukemia. *Journal of Oncology*.

[15] Yoshihara K., Shahmoradgoli M., Martínez E. (2013). Inferring tumour purity and stromal and immune cell admixture from expression data. *Nature Communications*.

[16] Thorsson V., Gibbs D. L., Brown S. D. (2018). The immune landscape of cancer. *Immunity*.

[17] Malta T. M., Sokolov A., Gentles A. J. (2018). Machine learning identifies stemness features associated with oncogenic dedifferentiation. *Cell*.

[18] Zhang X., Klamer B., Li J., Fernandez S., Li L. (2020). A pan-cancer study of class-3 semaphorins as therapeutic targets in cancer. *BMC Medical Genomics*.

[19] (2018). *RC T. R: A Language and Environment for Statistical Computing*.

[20] Saeed S., Quintin J., Kerstens H. H. D. (2014). Epigenetic programming of monocyte-to-macrophage differentiation and trained innate immunity. *Science*.

[21] Cheng S. C., Quintin J., Cramer R. A. (2014). mTOR- and HIF-1*α*-mediated aerobic glycolysis as metabolic basis for trained immunity. *Science*.

[22] Chen L., Kostadima M., Martens J. H. A. (2014). Transcriptional diversity during lineage commitment of human blood progenitors. *Science*.

[23] Tamborero D., Rubio-Perez C., Muiños F. (2018). A pan-cancer landscape of interactions between solid tumors and infiltrating immune cell populations. *Clinical Cancer Research*.

[24] Becht E., Giraldo N. A., Lacroix L. (2016). Estimating the population abundance of tissue-infiltrating immune and stromal cell populations using gene expression. *Genome Biology*.

[25] Ford D. J., Dingwall A. K. (2015). The cancer COMPASS: navigating the functions of MLL complexes in cancer. *Cancer Genetics*.

[26] Yang W., Ernst P. (2017). SET/MLL family proteins in hematopoiesis and leukemia. *International Journal of Hematology*.

[27] Mueller D., García-Cuéllar M.-P., Bach C., Buhl S., Maethner E., Slany R. K. (2009). Misguided transcriptional elongation causes mixed lineage leukemia. *PLoS Biology*.

[28] Dawson M. A., Prinjha R. K., Dittmann A. (2011). Inhibition of BET recruitment to chromatin as an effective treatment for MLL-fusion leukaemia. *Nature*.

[29] Brzezinka K., Nevedomskaya E., Lesche R. (2020). Characterization of the menin-MLL interaction as therapeutic cancer target. *Cancers*.

[30] Zeng X., Pan D., Wu H. (2019). Transcriptional activation of ANO1 promotes gastric cancer progression. *Biochemical and Biophysical Research Communications*.

[31] Kim S. H., Park W. S., Chung J. (2019). SETD2, GIGYF2, FGFR3, BCR, KMT2C, and TSC2as candidate genes for differentiating multilocular cystic renal neoplasm of low malignant potential from clear cell renal cell carcinoma with cystic change. *Investigative and Clinical Urology*.

[32] Dawkins J. B. N., Wang J., Maniati E. (2016). Reduced expression of histone methyltransferases KMT2C and KMT2D correlates with improved outcome in pancreatic ductal adenocarcinoma. *Cancer Research*.

[33] Xia M., Xu L., Leng Y. (2015). Downregulation of MLL3 in esophageal squamous cell carcinoma is required for the growth and metastasis of cancer cells. *Tumor Biology*.

[34] Alam H., Tang M., Maitituoheti M. (2020). KMT2D deficiency impairs super-enhancers to confer a glycolytic vulnerability in lung cancer. *Cancer Cell*.

[35] Zhang X., Novera W., Zhang Y., Deng L.-W. (2017). MLL5 (KMT2E): structure, function, and clinical relevance. *Cellular and Molecular Life Sciences*.

[36] Xu B., Qin T., Yu J., Giordano T. J., Sartor M. A., Koenig R. J. (2020). Novel role of ASH1L histone methyltransferase in anaplastic thyroid carcinoma. *Journal of Biological Chemistry*.

[37] Ansari K. I., Kasiri S., Mishra B. P., Mandal S. S. (2012). Mixed lineage leukaemia-4 regulates cell-cycle progression and cell viability and its depletion suppresses growth of xenografted tumour in vivo. *British Journal of Cancer*.

