# Supplementary figures and images for: A Pan-Cancer Study of KMT2 Family as Therapeutic Targets in Cancer
Source: J Oncol. 2022 Jan 11;2022:3982226. doi: 10.1155/2022/3982226 (PMC8766195; doi:10.1155/2022/3982226)

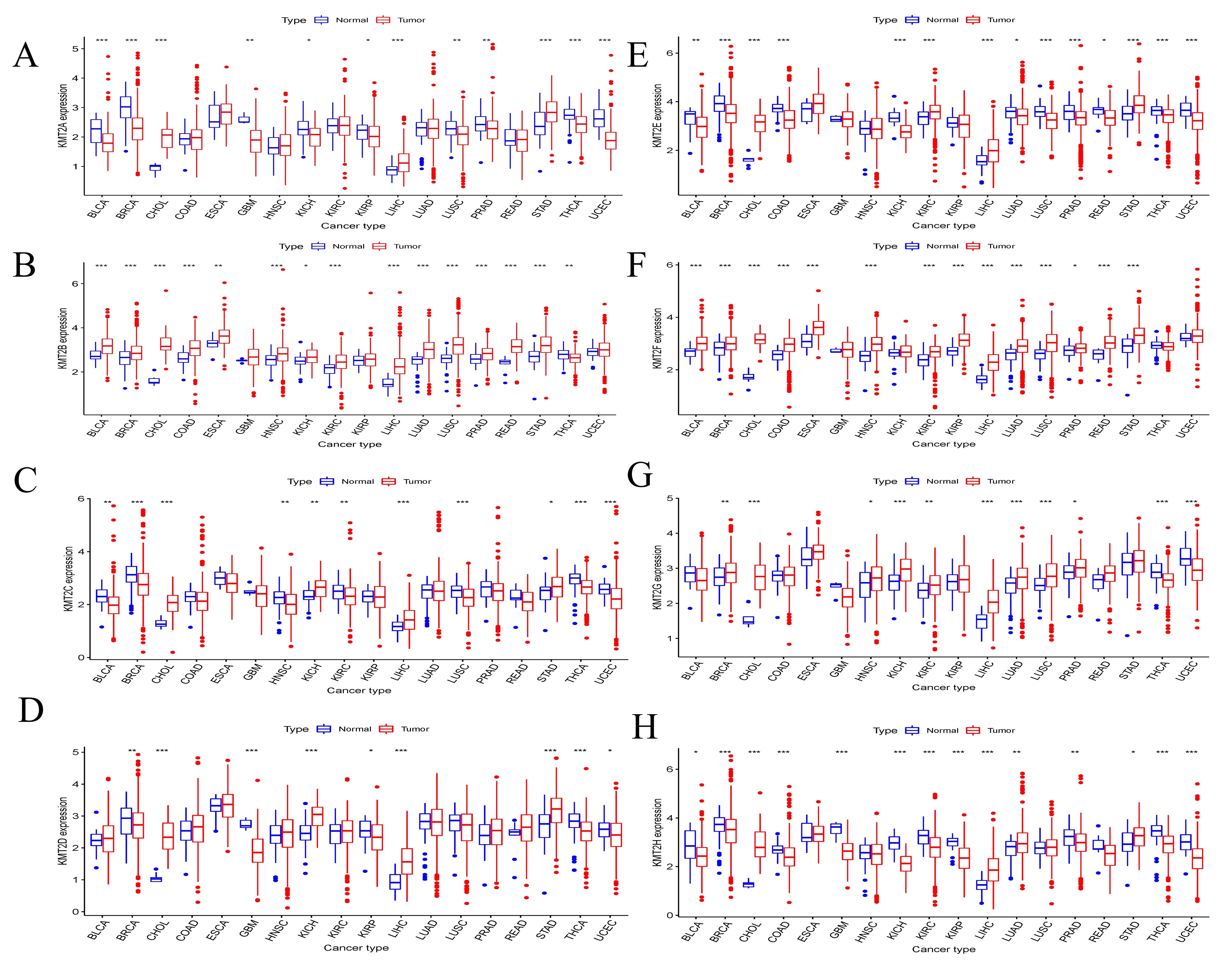

Supplement: Supplementary Materials — Supplementary Table 1: the 33 cancer types included in the TCGA pan-cancer data. Supplementary Figure 1: the levels of expression of KMT2 family members in all cancer types available in TCGA pan-cancer data. Supplementary Figure 2: overall survival in patients with renal clear cell carcinoma (KIRC) of KMT2 family members. Supplementary Figure 3: the expression of KMT2s in KIRC. s [file 3982226.f1.zip › 3982226.f1/Supplement F1.jpg]

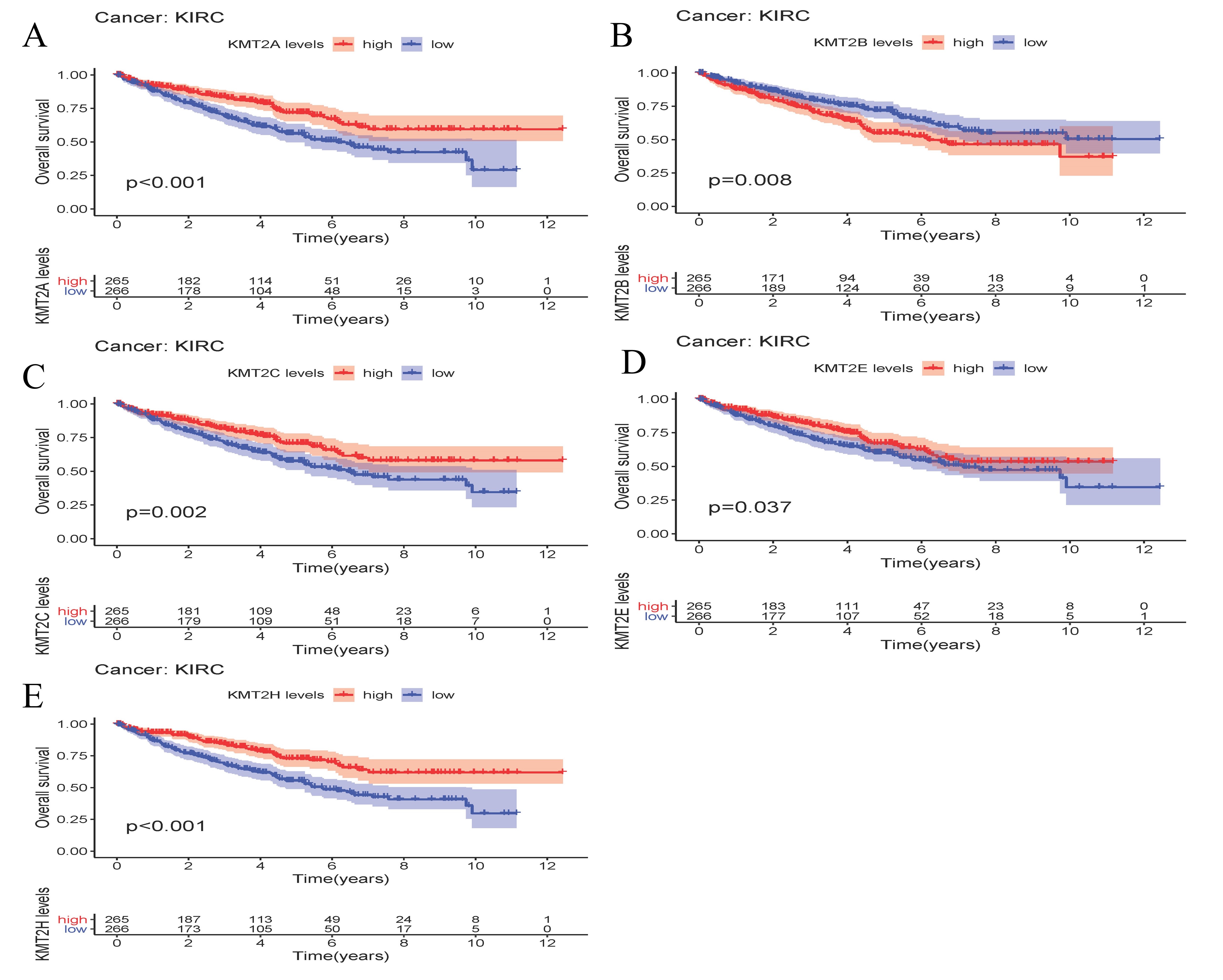

Supplement: Supplementary Materials — Supplementary Table 1: the 33 cancer types included in the TCGA pan-cancer data. Supplementary Figure 1: the levels of expression of KMT2 family members in all cancer types available in TCGA pan-cancer data. Supplementary Figure 2: overall survival in patients with renal clear cell carcinoma (KIRC) of KMT2 family members. Supplementary Figure 3: the expression of KMT2s in KIRC. s [file 3982226.f1.zip › 3982226.f1/Supplement F2.jpg]

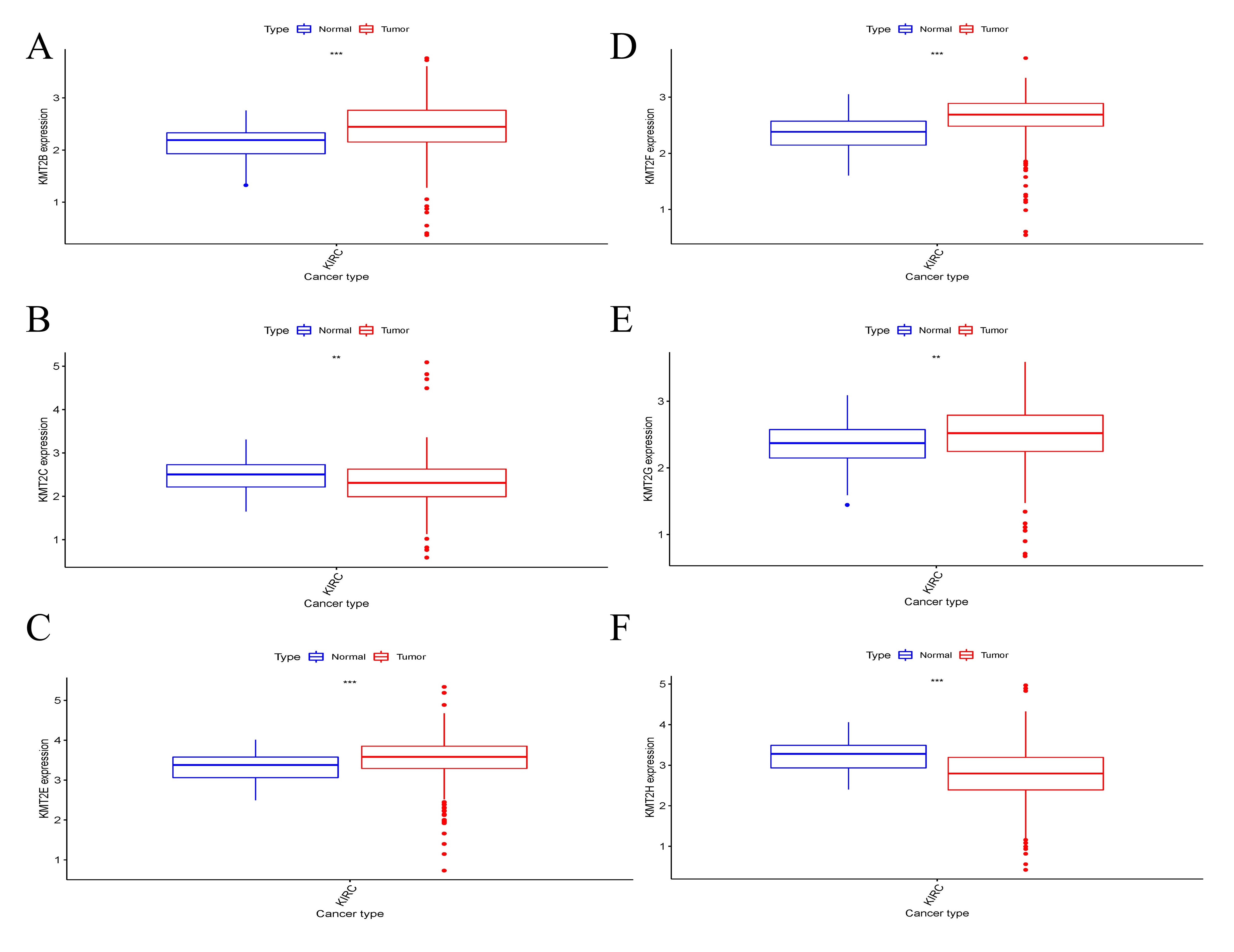

Supplement: Supplementary Materials — Supplementary Table 1: the 33 cancer types included in the TCGA pan-cancer data. Supplementary Figure 1: the levels of expression of KMT2 family members in all cancer types available in TCGA pan-cancer data. Supplementary Figure 2: overall survival in patients with renal clear cell carcinoma (KIRC) of KMT2 family members. Supplementary Figure 3: the expression of KMT2s in KIRC. s [file 3982226.f1.zip › 3982226.f1/Supplement F3.jpg]
